# Interventional Radiotherapy (Brachytherapy) for Nasal Vestibule: Novel Strategies to Prevent Side Effects

**DOI:** 10.3390/jcm12196154

**Published:** 2023-09-24

**Authors:** Bruno Fionda, Francesco Bussu, Elisa Placidi, Enrico Rosa, Valentina Lancellotta, Claudio Parrilla, Tiziano Zinicola, Martina De Angeli, Francesca Greco, Mario Rigante, Mariangela Massaccesi, Maria Antonietta Gambacorta, Luca Indovina, Marco De Spirito, Luca Tagliaferri

**Affiliations:** 1 U.O.C. Radioterapia Oncologica, Dipartimento di Diagnostica per Immagini, Radioterapia Oncologica ed Ematologia, Fondazione Policlinico Universitario “Agostino Gemelli” IRCCS, 00168 Roma, Italy; bruno.fionda@policlinicogemelli.it (B.F.); tiziano.zinicola@guest.policlinicogemelli.it (T.Z.); martina.deangeli@guest.policlinicogemelli.it (M.D.A.); mariangela.massaccesi@policlinicogemelli.it (M.M.); mariaantonietta.gambacorta@policlinicogemelli.it (M.A.G.);; 2 Divisione di Otorinolaringoiatria, Azienda Ospedaliero Universitaria, 07100 Sassari, Italy; fbussu@uniss.it; 3 Dipartimento di Medicina, Chirurgia e Farmacia Università di Sassari, 00168 Sassari, Italy; 4 U.O.S.D. Fisica Medica e Radioprotezione, Dipartimento di Diagnostica per Immagini, Radioterapia Oncologica ed Ematologia, Fondazione Policlinico Universitario Agostino Gemelli IRCCS, 00168 Rome, Italy; francesca.greco@policlinicogemelli.it (F.G.); luca.indovina@policlinicogemelli.it (L.I.); 5 Dipartimento di Neuroscienze, Sezione di Fisica, Università Cattolica del Sacro Cuore, 00168 Rome, Italy; 6 U.O.C. Otorinolaringoiatria, Dipartimento di Scienze dell’Invecchiamento, Neurologiche, Ortopediche e della Testa-Collo, Fondazione Policlinico Universitario Agostino Gemelli IRCCS, 00168 Rome, Italy; 7 Istituto di Radiologia, Università Cattolica del Sacro Cuore, 00168 Rome, Italy; 8 Fondazione Policlinico Universitario Agostino Gemelli, IRCCS, 00168 Rome, Italy

**Keywords:** nose vestibule, nasal vestibule, interventional radiotherapy, brachytherapy, radiotherapy

## Abstract

Interventional radiotherapy (brachytherapy) has become the new therapeutic standard in the management of early stages nasal vestibule tumors; in fact it allows for high local control rates and low toxicity profiles. However, since more and more patients will receive interventional radiotherapy (brachytherapy) as primary treatment, it is desirable to implement novel strategies to reduce the dose to organs at risk with the future aim to result in further lowering long-term side effects. Materials and methods: We were able to identify two different strategies to reduce dose to the treatment volume, including the implantation technique (the implant can be interstitial, endocavitary or mixed and the catheters may be placed either using the Paris system rules or the anatomical approach) and the dose distribution within the implant (the most commonly used parameter to consider is the dose non-uniformity ratio). We subsequently propose two novel strategies to reduce dose to organs at risk, including the use of metal shields for fixed organs as in the case of the eyes and the use of a mouth swab to push away mobile organs, such in the case of the mandible. We used two different algorithms to verify the values namely the TG-43 and the TG-186. Results: We provided an accurate literature review regarding strategies to reduce toxicity to the treatment volume, underlining the pros and cons of all implantation techniques and about the use dose non-uniformity ratio. Regarding the innovative strategies to reduce the dose to organs at risk, we investigated the use of eye shielding and the use of swabs to push away the mandible by performing an innovative calculation using two different algorithms in a series of three consecutive patients. Our results show that the dose reduction, both in the case of the mandible and in the case of eye shielding, was statistically significant. Conclusion: Proper knowledge of the best implantation technique and dose non-uniformity ratio as highlighted by existing literature is mandatory in order to reduce toxicity within the treatment volume. With regard to the dose reduction to the organs at risk we have demonstrated that the use of eye shielding and mouth swab could play a pivotal role in clinical practice; in fact, they are effective at lowering the doses to the surrounding organs and do not require any change to the current clinical workflow.

## 1. Introduction

Nasal vestibule (NV) tumors are usually considered a very rare entity. In a large retrospective series from Denmark, covering the entire population for a period of ten years, the estimated annual incidence was found to be 0.32 and 0.41 per 100,000 habitants, However, a recent update of the same national database pointed out that underreporting, caused by misdiagnosis as skin carcinoma, could be a confounding factor [1,2].

In general, the incidence of nasal vestibule carcinomas is probably underestimated, not only because of such misdiagnosis and misclassification, but also for the absence of clear radiologically identifiable boundaries and of a specific WHO topographic code to obtain incidence data from cancer registries. A plane passing tangential to the piriform opening has been proposed as the posterior boundary of the nasal vestibule, allowing for the assignment of a specific topographic code [3].

Moreover, the specific patterns of spread of NV cancers make the UICC/AJCC T classification criteria, in common with ethmoid and nasal cavity proper, inadequate for prognostic stratification, Wang’s classification specifically designed for nose vestibule, and dating back to 1976, has been demonstrated to predict survival better [4]. Wang’s criteria have been re-used for the novel Bussu T classification system, which has been, recently, preliminarily evaluated in a large multicentric series with very promising results [5].

In the treatment phase, surgery is very effective from an oncological point of view, as negative margins can be easily obtained in the ablative phase. However, nasal vestibule lies in the midface and is the most exposed and noticed part of the whole body, where light and human eye always fall: minimal imperfections, scars, and deformity, as those deriving from a surgical resection, have the highest esthetical and social impact.

Considering that ablative surgery in nose vestibule SCC, typically growing along and between the cartilages, requires almost always a “through” resection, it is impossible to faithfully restore the complex pattern of relieves and hollows created by the nose cartilages and underlying maxillary and nasal bones by reconstructive surgery. Local flaps are not enough, free flaps are too much and do not have a definitive impact in NV, scars and color mismatches are most easily noticed: preservation of the anatomy and of the cartilaginous framework remains the most successful option from a cosmetic point of view [6,7,8].

Moreover, considering that nose cartilage itself generally tolerates radiation very well (if the trophic support by perichondrium is preserved), the preservation of the nose tip framework, with consequently markedly better cosmetic results, reported for high dose rate (HDR) interventional radiotherapy (IRT—Brachytherapy) when compared to surgery, and can be easily explained. Also, EBRT is considered a valid alternative option from an esthetic point of view and is currently probably the most frequently recommended primary treatment in Western countries [1,7,9].

Nevertheless, both oncological [10] and functional outcomes of IRT are superior to EBRT in case of nasal vestibule malignancies.

For these reasons, IRT is becoming the new standard treatment for nasal vestibule carcinomas [11].

Further clear advantages of IRT are easy exposure of the clinical target volume, easy tube placement also under local anesthesia (elderly/frail patients); no vital structure to preserve in close proximity; and possibility for a very high dose and/or boost.

However, as always when dealing with curative doses of radiotherapy, toxicity is a relevant issue affecting treatment planning, and even if vital structures constituting the major concerns of head and neck radiation oncology, as central nervous system and carotid axis, are well distant from the CTV; however, the eye(s) can be a relevant problem in bulky lesions with a cranial spread. Considering that a very high dose is often delivered, with the typical dose inhomogeneity of IRT, this also toxicities on soft tissues and on skin and mucosal surfaces, which can be very relevant. For this reason, different strategies could be considered to reduce the dose both within the volume to treat and in the nearby tissues and organs at risk.

With regard to the volume to treat, we may identify at least two different relevant points:

Implantation technique: the implant can be interstitial or endocavitary, using nasal packing or personalized molds for stabilization; a mixed approach including simultaneous endocavitary, interstitial and/or contact (as in the skin primaries) delivery can also be followed in tricky cases. The interstitial implantation can follow the principles of the traditional Paris system or of the novel anatomical implantation [11].

Dose distribution within the implant: one the most commonly used parameters to consider is the dose non-uniformity ratio (DNR), which consists of evaluating the relationship between the volume receiving 1.5 times the prescribed dose.

Concerning the dose reduction to organs at risk, we propose two different approaches: 

Use of shields for fixed organs: in the case of the eyes (lens), it is possible to use metal shielding to reduce the dose received.

The use of a personalized mouth swab to push away mobile organs: in the case of surrounding anatomical structures such as the oral cavity, buccal mucosa, inferior lip, and mandible, it is possible to use a mouth swab to push away these structures.

The aim of this article was to review the most rational strategies used by our group to reduce the toxicity of IRT for primary NV carcinomas, comparing and compounding them with the current literature concerning implant techniques and dose distribution.

## 2. Materials and Methods

Three consecutive patients affected by NV cancer cases were used for the purpose of this study, and a CT scan was obtained with a slice thickness of 0.625 mm on a Discovery CT590RT CT scanner (GE Medical System). An Oncentra Brachy treatment planning system (TPS v.4.6.2 Elekta, Sweden) was used for organs at risk (OARs), contouring, and planning; the prescribed dose was 44 Gy and 2 fractions were delivered daily, all patients provided informed consent before treatment. In interstitial head and neck IRT, sparing of normal tissues and OARs, is critical for the specific region of the treatment. For such reasons in this report, we referred to absorbed doses by eye lens and mandible in the treatment. In order to determine the D2cc for the mandible and the D0.01cc for the lens, treatment plans were performed, varying the dose calculation algorithm, the position of the mandible and the presence of eye protections. This analysis was performed as a practical approach to reduce the dose to OARs for the NV treatment plan. The mean CTV volume was 11.35 cc and the lesions were all staged as T2 according to the Bussu classification.

**Figure 1:**
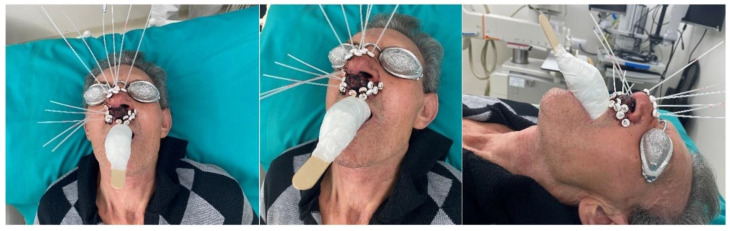
Patient set-up for treatment delivery.

**Figure 2:**
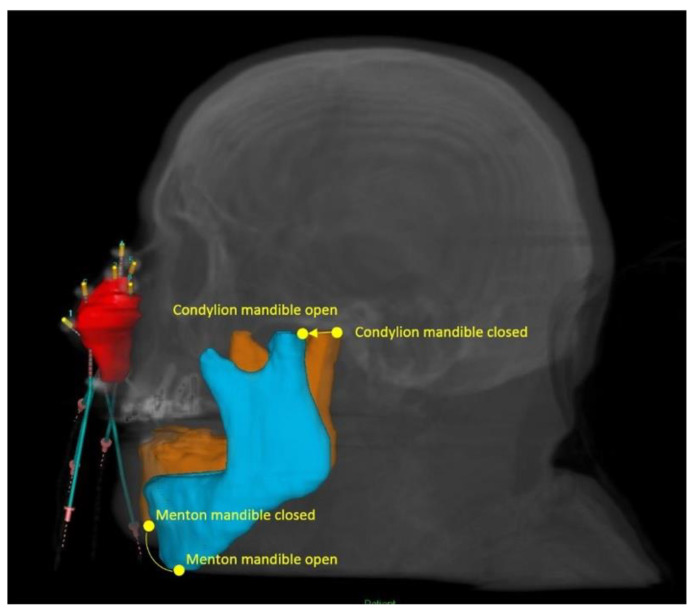
Digital reconstruction of the CTV with the activated catheters. The condylion and the menton mandible points are highlighted for the two configurations: closed and at the maximum mandible opening (MMO).

Eyes, lens, bones and mandible were contoured. A second CT scan was acquired using the same interstitial nasal implant with eye protection and a spacer to distance the mandible for the implant region (Figure 1). The CT scans were registered and the contours were performed on the first CT without eye shielding and protection. Figure 2 shows the CTV contour and the activation of the catheters in one of the patients. Treatment plans were performed by an experienced planner. In the first part of this study, dose calculations for the iridium-192 high-dose rate (HDR) source were performed with the TG-43 formalism [12]. The patients enrolled in this study were asked to open their mouth as much as possible by means of the use of a personalized mouth swab filled with water. The angle in the sagittal plane between the mandible open with the spacer (maximum mandible opening, MMO) and the closed mandible was determined, as shown in Figure 2, and the translation distance from the jaw attachment of the two positions was measured in the 3D-space. 

**Figure 3:**
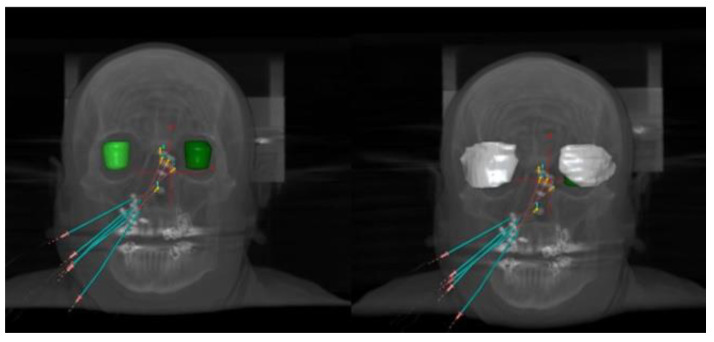
CT of a patient with focus on eyes with and without shielding (with—left/without—right).

In a second step, the calculations were performed with the TG-186 formalism (ACE, Advanced Calculation Engine, Elekta) [13] by keeping the same dwell positions and activation times. In order to calculate the dose reduction to the OARs due to the metal eye shielding, the protections were contoured in the second CT and copied to the first one; the shielding was defined as lead material with a uniform density of 11.1 g/cm^3^ for the TG-186 calculations as shown in Figure 3. The mandible with the spacer was contoured in the second CT and copied to the first one to evaluate the distance in translation and rotation from the closed mandible. Bones and mandibles were contoured and assigned to “cortical bone” with a uniform density value of 1.920 g/cm^3^. The eyes and lens were assigned a material of “lens” and a HU-based density. The external body was defined as soft tissue with an HU-based value of density, while the existing artifact caused by tooth implants was contoured and assigned to uniform cortical bone for teeth and uniform soft tissue for the remaining region. A 3 cm thick air layer surrounding the body was considered and assigned to a uniform air density of 0.001 g/cm^3^ to take into account the air around the patient’s body.

All data were expressed as mean with SD, and analyzed by a one-way t-student test.

## 3. Results

The analysis of the data presented in this study highlights significant findings regarding the mean angle and distance measurements. The mean angle between the menton mandible point closed and at MMO, determined to be 13° ± 5°, provides crucial insights into the spatial relationship between these points. Additionally, the mean distance between the condylion points, measuring 4.3 ± 1.9 cm, sheds light on the anatomical considerations of this study.

Table 1 demonstrates the mean dose reduction observed at two points, amounting to 14% and 55%, respectively. These reductions signify the effectiveness of the approach in minimizing radiation exposure.

Table 2 showcases the outcomes for the D2cc of the mandible, computed using both TG-43 and TG-186 algorithms. Notably, both algorithms indicate a significant reduction of over 41%, attributed to the increased distance of the mandible from the implant. This finding underscores the importance of algorithm selection in treatment planning.

In Table 3, the results for the eyes and eye lenses are presented. Specifically, D0.01cc and D2cc values for these organs are presented both with and without shielding. It’s worth noting that, based on the analysis, the TG-186 algorithm was exclusively utilized in this study.

The application of the TG-186 algorithm resulted in remarkable dose reductions, with an 82% decrease for the right lens and a 66% reduction for the left lens. Furthermore, a noteworthy reduction between 22% and 24% was observed for the eyes, underscoring the algorithm’s efficacy in safeguarding these sensitive structures.

An encouraging aspect of this study is that the implementation of these protective measures did not necessitate any workflow modifications. Consequently, no additional time was required to integrate these devices into the treatment strategy.

To assess the impact on patient well-being, all three patients underwent regular assessments for acute toxicity at 1-, 3-, and 6 months post-treatment. Encouragingly, none of the patients reported significant toxicity related to the eyes or mandible. Furthermore, there were no notable acute adverse events documented in relation to the lower lip and mouth floor. These findings suggest the safety and feasibility of the employed strategy in minimizing radiation-related complications.

## 4. Discussion

In our study we were to obtain a statistically significant reduction in terms of dose to OARs for lens and for the mandible using personalized devices during the treatment delivery with a safe and practical approach, which did not require any additional time consumption for the delivery.

Modern interventional radiotherapy for H&N cancers is characterized by several technological improvements, such as image guidance and dose modulation, which, when combined with the favorable dosimetric properties of the radioactive sources (rapid dose fall-off), allow the delivery of high doses to the target with a maximum sparing of the surrounding tissues and organs at risk [14].

The most commonly reported side effects for combined mold-based intracavitary and the interstitial PDR brachytherapy technique are nasal crusts (66.1%), ulcers (16.1%), chondroradionecrosis (9.7%) and epistaxis (6.5%) [15]. With regard to chondroradionecrosis, a much lower incidence (range 0–4%) is reported in the interstitial HDR approach, suggesting that both the use of image guidance and a learning curve over time, as well as the new anatomical implantation for NV carcinomas, allow for better results [10,16].

Several authors have recently proposed that, for head and neck tumors, the concept of “in-field” toxicity [17,18], and therefore, this could be useful to distinguish “in-field” and “out-of-field” toxicity that could be prevented using different approaches. 

Interestingly, some authors have recently proposed to group possible toxicities in head and neck patients receiving radiotherapy into six so-called toxicity domains, which include swallowing, salivary, mucosal, speech, pain and a general domain. Each of these domains is associated with specific side effects, namely dysphagia for the swallowing domain, xerostomia for the salivary domain, mucositis for the mucosal domain, hoarseness for the speech domain, oral/jaw pain for the pain domain and weight loss for the general domain. In particular, the authors were able to identify 14 OARs involved in one or more toxicities and, more specifically, the oral cavity was associated with five toxicity domains out of six [19].

The most important late side effect recorded in clinical studies about IRT in NV is represented by chondronecrosis associated with septal and/or alar perforations. This adverse event is more commonly reported in the case of interstitial implants compared to the endocavitary/mould approach. It has been described that this is probably due to the fact that disrupting the perichondrium, the layer that actually feeds the cartilage, is the leading cause bringing about subsequent chondronecrosis. Therefore, careful preservation of the perichondrium and of the cartilage should be meticulously pursued. When performing the implant, the plastic tube routes should be anatomical, which means along the subperichondral planes. The geometry of the NV implant therefore should be predetermined as usually performed according to the Paris system rules but should be personalized to the noses anatomy of the patients. Sometimes, a combined interstitial and intracavitary implant can be considered in cases of tumor extending posteriorly into the nasal cavities that could therefore benefit from a dosimetric point of view from a further dose modulation [20].

The Dose non-uniformity ratio (DNR) is the ratio of the high-dose volume to the reference dose volume, and the commonly accepted definition high-dose volume is the volume that receives 1.5 times or more of the prescribed dose [21].

Available recommendations for IRT in H&N cancers underline that DNR should be below 0.35 [22]. Available dosimetric data published in a retrospective series showed that higher DNR values are not associated with an increase in terms of adverse events [16]. This finding suggests that, probably in the case of NV, the small volumes of treatment, combined with the anatomical implantation technique, could allow for higher DNR values with no additional toxicity, as long as the perichondrium and the cartilages are not pierced. An additional element to consider is that the DNR should also take into account the BED_10_ of the different fractionations reported in the literature (range from 58 Gy to 66 Gy); lower values of BED_10_ higher values DNR could be acceptable.

Current available data about constraints for the anterior segment of the eye include, for conventional fractionations, Lens D_max_ < 4 Gy, Cornea D_max_ < 40 Gy and Lacrimal Gland D_max_ < 40 Gy. 

With regard to the dose to ocular structures, a recent publication concluded that the decision to use an ocular shielding during facial IRT procedures should be made on a patient-by-patient basis, and based on model-based calculation methods recommended by TG186 [23].

With regard to the oral cavity, the V30 Gy < 73% (limiting the D_mean_ to uninvolved oral cavity) and lips should not exceed a D_mean_ of 30 Gy [24,25]. Interestingly, the head, neck and skin GEC-ESTRO working group has recently issued some recommendations with regard to dose constraints, and found that ORN correlates with Total Physical Dose to Mandible_2cm3_; in fact, patients receiving total physical doses greater than 61 Gy had a 20-fold increased risk of ORN [26].

Several variables have been investigated and associated with the development of mandibular osteoradionecrosis (ORN) in H&N patients receiving radiotherapy; among them, we may report patient-related factors such as smoking, dental hygiene, alcohol consumption and other comorbidities, as well as treatment-related factors. With regard to the treatment-related factors in recent years, dose–volume correlations of the irradiated mandible has gained particular attention. D_mean_ has been extensively studied in a large cohort of patients affected by head and neck cancer and treated by external beam radiotherapy as a possible parameter to consider in the planning because it seems reasonable in the case of the mandible, which could be assumed to be a parallel organ. However, no accepted dose threshold was proposed, and the author concluded to keep doses as low as possible to reduce the risk of ORN [27].

The mandible may have six degrees of freedom [28]; however, for dosimetric purposes, it is important to underline the roto-translational movement on the sagittal plane [29]. More specifically, in the first stage, namely up to 10°, the main movement is basically represented by rotation; further rotation of the mandible is characterized by rotation accompanied by dislocation in the antero-inferior direction [30].

With regard to the treatment of ORN, there are different strategies that can be either conservative or include a surgical intervention; among the conservative options, it is possible to consider local irrigations, systemic antibiotic, avoidance of irritants (tobacco, alcohol, denture use), oral hygiene instruction and, in selected cases, even hyperbaric oxygen [31].

The first dedicated classification system was issued by Wang in 1976 [32]. However, over the years, different staging systems have been used to classify NV cancers, including TNM and, even simpler, the size of the primary lesion [3].

Tumor size is a major indicator after irradiation, both for local and regional control [33,34]. So, whereas most authors agree that IRT is the treatment of choice for early lesions, there is less evidence about the optimal management for more advanced tumors; current indications include using RT for favorable T4 and consider a combination strategy (surgery and RT) for very advanced T4 [35,36].

More recently, a new staging system has been proposed that takes into account the main natural ways of diffusion: posterior, along the septal cartilage; lateral, between the alar and triangular cartilage directly on the skin; and inferior, on the upper lip. In a large multicenter retrospective analysis, this new classification has been shown to better stratify tumoral lesions, even compared to Wang’s system. One of the key points of this new system is the definition of NV, not only as the area just inside the nostril that leads into the nasal cavity [37], but as the area that redefines its anatomical limits using the plane passing through the piriform opening [11]. In fact, NV tumors are completely different from nasal cavity tumors due to late involvement of the bone, with poor prognostic implications and very early skin involvement.

The lymphatic drainage of the NV might be bilateral and the typical lymph nodes involved are submandibular, facial and preauricular [38]; with regard to the management, in case of uninvolved regional nodes, elective treatment is not considered necessary [39,40]. 

When analyzing the functional outcome of surgery, external beam radiotherapy and IRT, also in terms of cytological findings, shows that the results present significant improvements in patients treated by IRT.

Another important point to consider is the cosmetic assessment of patients after IRT and, also, in this case, by using image guidance, the patient-assessed cosmetic satisfaction is as high [41].

With regard to the dose rate, the data reported suggests that HDR IRT allows for improved results compared to LDR [42,43].

Since NV tumors are relatively rare and a multidisciplinary approach is mandatory for the management of these patients, it would be desirable to concentrate the cases into a high-volume center with a dedicated team and specific expertise.

Even though, in most retrospective series toxicity profiles are favorable, making IRT for NV a well-tolerated treatment, it is important to implement new strategies aimed at further reducing the dose to nearby tissues with the intent to reduce or even prevent any adverse event from occurring. This effort will have a more and more relevant role as IRT is becoming the first choice in the management of NV cancers and higher volumes of patients will receive this treatment. 

## 5. Conclusions

Interventional radiotherapy (brachytherapy) represents an optimal therapeutic choice in the management of early stages (T1/2 according to Wang, T1/2/3 according to Bussu) of nasal vestibule tumors. The toxicity profile in retrospective series is usually low, but implementing novel strategies to reduce the dose to organs at risk could result in further lowering of long-term side effects and, therefore, these new proposals are desirable and need additional evaluation in larger series.

## Figures and Tables

**Table 1:** Mean dose results for condylion and menton points. *p* value calculated with t-student test is shown, values marked with * are statistically significant.

Mandible Points	Dose for Closed Mandible (Gy and SD)	Dose for Open Mandible (Gy and SD)	Dose Reduction	*p* Value
Condylion TG-43	0.07 ± 0.03	0.06 ± 0.03	−14%	0.6125
Menton TG-43	0.11 ± 0.03	0.05 ± 0.02	−55%	0.0059 *

**Table 2:** Mean D2cc and SD of the mandible calculated with the TG-43 and the TG-186 algorithms for the mandible at MMO and without spacer. A total reduction of more than 40% is found for both algorithms, *p* value calculated with t-student test is shown, (* for statistically significant).

	Mandible with Spacer at MMO (Gy)	Mandible without Spacer (Gy)	Dose Reduction	*p* Value
D2cc Mandible TG-43	0.30 ± 0.06	0.51 ± 0.18	−41%	0.04 *
D2cc Mandible TG-186	0.27 ± 0.35	0.46 ± 0.56	−42%	0.02 *

**Table 3:** Mean dosimetric constraints and SD for the eyes and eye lenses. A dose reduction is found with the TG-186 algorithm when a shielding is used. *p* value calculated with t-student test is shown, value marked with * are statistically significant.

	With Eye Shielding (Gy) and SD	Without Eye Shielding (Gy) and SD	Dose Reduction	*p* Value
D0.01cc R lens TG-186	0.08 ± 0.06	0.44 ± 0.06	−82%	0.0003 *
D0.01cc L lens TG-186	0.15 ± 0.11	0.44 ± 0.10	−66%	0.0023 *
D2cc R Eye TG-186	0.28 ± 0.13	0.37 ± 0.11	−24%	0.277
D2cc L Eye TG-186	0.26 ± 0.11	0.33 ± 0.11	−22%	0.282

## Data Availability

Not applicable.
